# In Vivo Measurement of Tidal Volume During Non-invasive Respiratory Support by Continuous-Flow Helmet CPAP

**DOI:** 10.1007/s10439-024-03545-6

**Published:** 2024-06-17

**Authors:** A. LoMauro, A. De Luca, P. Scarpazza, A. Aliverti

**Affiliations:** 1 Dipartimento di Elettronica, Informazione e Bioingegneria, Politecnico di Milano, P.zza L. da Vinci, 32, 20133 Milan, Italy; 2 Pneumology Unit, Ospedale Civile, Vimercate, Milan Italy

**Keywords:** Non-invasive respiratory support, H-CPAP, Tidal volume, Optoelectronic plethysmography, Posture, Flow, PEEP

## Abstract

Recently, the interest in the Helmet interface during non-invasive respiratory support (NIRS) has increased due to the COVID-19 pandemic. During NIRS, positive end-expiratory pressure (PEEP) can be given as continuous positive airway pressure (CPAP), which maintains a positive airway pressure throughout the whole respiratory cycle with Helmet as an interface (H-CPAP). The main disadvantage of the H-CPAP is the inability to measure tidal volume (V_T_). Opto-electronic plethysmography (OEP) is a non-invasive technique that is not sensitive to gas compression/expansion inside the helmet. OEP acquisitions were performed on 28 healthy volunteers (14 females and 14 males) at baseline and during Helmet CPAP. The effect of posture (semi-recumbent vs. prone), flow (50 vs. 60 L/min), and PEEP (0 vs. 5 vs. 10 cmH_2_O) on the ventilatory and thoracic-abdominal pattern and the operational volumes were investigated. Prone position limited vital capacity, abdominal expansion and chest wall recruitment. A constant flow of 60 L/min reduced the need for the subject to ventilate while having a slight recruitment effect (100 mL) in the semi-recumbent position. A progressive increasing recruitment was found with higher PEEP but limited by the prone position. It is possible to accurately measure tidal volume during H-CPAP to deliver non-invasive ventilatory support using opto-electronic plethysmography during different clinical settings.

## Introduction

The respiratory system supplies the body with oxygen and removes carbon dioxide. If either of these two functions fails, respiratory failure occurs leading to hypoxemia (arterial partial pressure of oxygen below 60 mmHg) or hypercapnia (partial pressure of carbon dioxide higher than 45 mmHg) [1–4]. A direct relationship between the degree of hypoxemia and mortality is known [5].

During the peak of the COVID-19 pandemic, the occupancy bed rate of the intensive care unit exceeded 100%, and the clinicians had to opt for non-invasive respiratory  support (NIRS) as an extrema urgent action to save patients. The role of NIRS in the management of acute hypoxemic respiratory failure (AHRF) is still unclear but evolving. Improved oxygenation combined with the protection of the lung produced by NIRS may avoid endotracheal intubation and prevent complications associated with invasive mechanical ventilation. Spontaneous breathing in patients with lung injuries, though, presents the risk of lung damage induced by excessively large tidal volumes (P-SILI). The risk of P-SILI can be reduced by applying a single level of continuous positive airway pressure (CPAP) during the entire respiratory cycle [5]. CPAP improves oxygenation, increases end-expiratory volume, contributes to lung protection and may increase lung compliance.

The interest in helmet as an interface was renewed during the COVID-19 pandemic [6]. The helmet has several advantages over the most traditional oro-nasal and face masks. It is well tolerated for the absence of complications related to ulcer formation. Air leaks are smaller during high levels of positive end-expiratory pressure (PEEP) [7] and prolonged treatment without interruption is allowed [8]. The main disadvantage of the helmet is the inability to measure tidal volume due to the gas compression/expansion inside the helmet. Opto-electronic plethysmography (OEP) is a non-invasive validated method that measures breathing volume without instrumenting the airways.

This work aimed to quantify the volume variations during Helmet CPAP (H-CPAP) using OEP on healthy subjects. To simulate clinical settings, we have evaluated the effects of prone and semi-recumbent positions, two levels of continuous flow and three levels of positive end-expiratory pressure on the ventilatory pattern and the operational volumes.

## Materials and Methods

### Opto-Electronic Plethysmography (OEP)

**Fig. 1:**
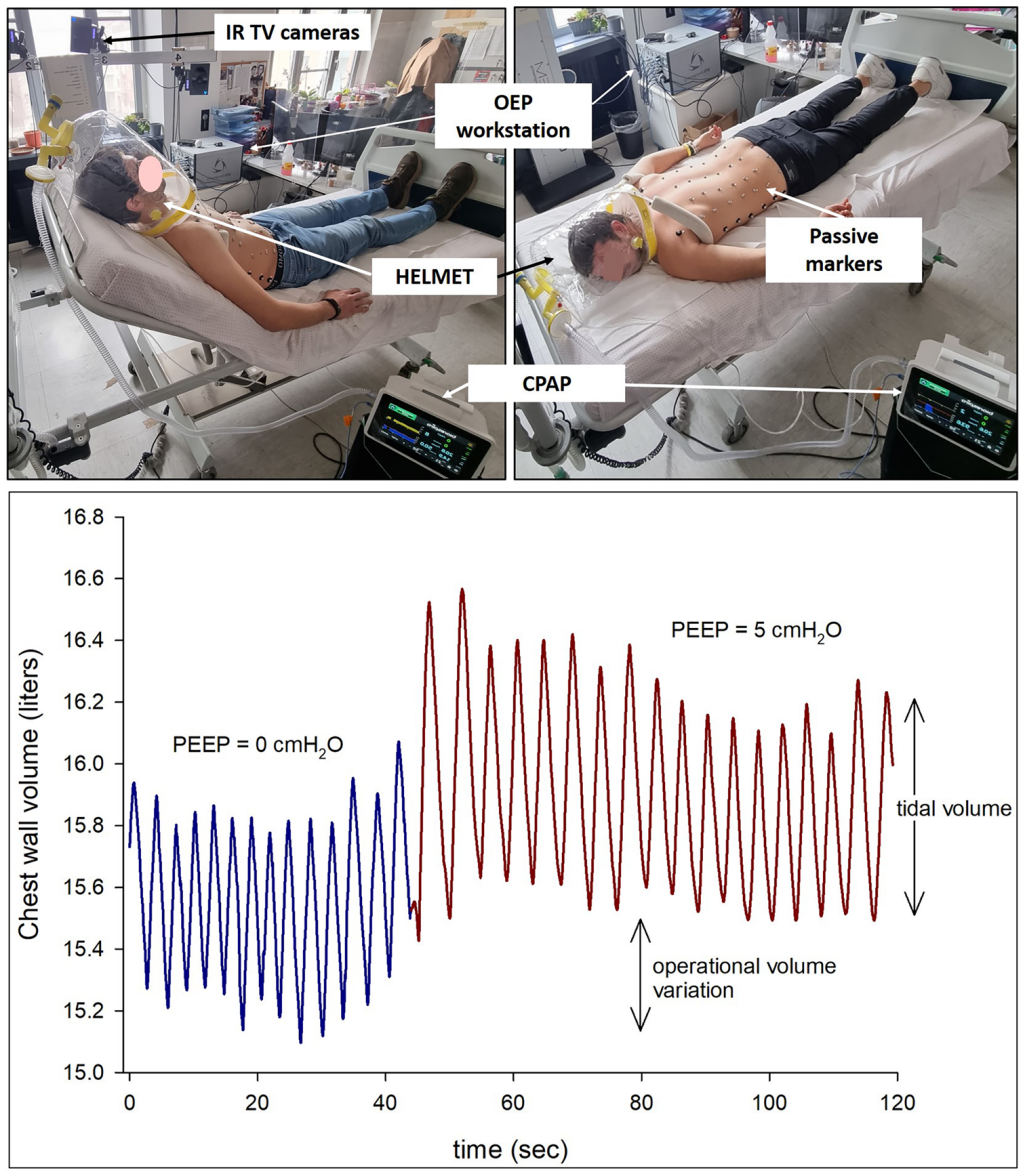
Experimental set-up of a subject wearing the passive markers on his thorax and the helmet in semi-recumbent and prone attached to the CPAP during opto-electronic plethysmography acquisition. The photos are published with the written permission of the subjects. In this study, acquisitions were performed with 8 infrared cameras and 52 markers were used, placed at precise points on the front and back of the subjects in the semi-recumbent and prone position, respectively (top left panels) [9, 10]. Starting from these 3D coordinates in association with dedicated geometrical models, the system provides the volume enclosed. An exemplificative trace of chest wall volume (top right panel) during the transition from zero end-expiratory pressure (blue) to a positive end-expiratory pressure of 5 cmH_2_O (red)

OEP system (OEP System; BTS, Milan, Italy [9–11]) is a motion analysis system based on infra-red TV cameras that provides the 3D coordinates of passive reflective markers put on the chest wall according to anatomical points. The system is calibrated a priori using a calibration object (i.e., three perpendicular axes with markers at a fixed distance) and is patient independent. Thanks to the Gauss’s theorem combined with dedicated geometrical models it is possible to computes the volume enclosed by the reflective markers placed on the chest wall. The Gauss’s theorem states that the surface integral of a vector field over a closed surface equals the volume integral of the divergence over the region enclosed by the surface. The geometrical models define the closed surface, being triangles with the markers as vertexes. OEP provides accurate measurements of the tidal volume and of the variations of the absolute volume (i.e., the operational volumes, Fig. 1) of the chest wall and its compartments: the rib cage and the abdomen. The volume signal does not require basic signal processing. The breaths are identified on the volume by selecting the end-inspiration (i.e., local maxima volume point) and end-expiration (i.e., local minima volume point) times. The complete ventilatory and thoraco-abdominal patterns are then computed on a breath-by-breath base. More in detail, the respiratory rate is the number of breaths taken per minute. The tidal volume is the difference between the volume at end-inspiration and end-expiration (Fig. 1). The minute ventilation is the product of the respiratory rate and tidal volume and it is the volume of gas inhaled per minute.

### Protocol of Acquisition

The research protocol was approved by the local research Ethics Committee of Politecnico di Milano (Decision n. 09/2023) according to the declaration of Helsinki. All subjects signed a written informed consent form. Subjects were recruited on a voluntary base among friends, colleagues or students of the authors. Inclusion criteria: absence of acute respiratory infection; absence of chronic respiratory disease; absence of airway deformation; no obesity (BMI < 25 kg/cm^2^) and no claustrophobia.

Each subject was placed on a reclining bed, in a semi-recumbent 45° position and then in a prone position. In the semi-recumbent 45° position, 52 markers were placed from the clavicles to the iliac crests [10]. In the prone position, 47 markers were placed from the C7 vertebrae to the posterior gluteal line [9] (Fig. 1).

The configuration of H-CPAP was: a constant flow (Biorespira, ibd, Mantova, Italy) of fresh gas (at room air) through the helmet (StarMed CaStar Intersurgical spa, Mirandola (MO), Italy) that was dispersed in ambient air through a positive end-expiratory pressure (PEEP) valve connected to the expiratory helmet port. The helmet was specifically designed for ventilation in prone/supine positions.

Once the subject became familiar with the position and the instrumentation, quiet breathing was acquired for 5 minutes at zero end-expiratory pressure (ZEEP) as a baseline and then the subject was asked to perform two vital capacities.

In both postures, four combinations of flow and PEEP were then applied to the subject breathing ambient air for approximately 5 min: (1) 50 L/min flow + 5 cmH_2_O PEEP; (2) 60 L/min flow + 5 cmH_2_O PEEP; (3) 60 L/min flow + 10 cmH_2_O PEEP; (4) 50 L/min flow + 10 cmH_2_O PEEP.

The operational volumes, and the ventilatory and thoraco-abdominal pattern were computed during the last minute of the acquisition.

### Statistical Analysis

All the statistical analyses have been performed in RStudio. The distribution of the data was first checked by the Kolmogorov-Smirnov test. When the analyzed parameters were normally distributed, a One Way Repeated Measures Analysis of Variance was performed. The Wilcoxon Matched Pairs test was used with posture or flow or PEEP as the independent factor for the parameters not normally distributed. The significance level was set at 95% (*p*-value < 0.05).

## Results

The study was performed on 28 healthy volunteers, 14 females and 14 males (median age: 24.9 years, median height: 174.6 cm, median weight: 69.8 kg; median BMI: 22.5 Kg/cm^2^).

### Postural Effect at Baseline: Semi-recumbent vs Prone Position

**Fig. 2:**
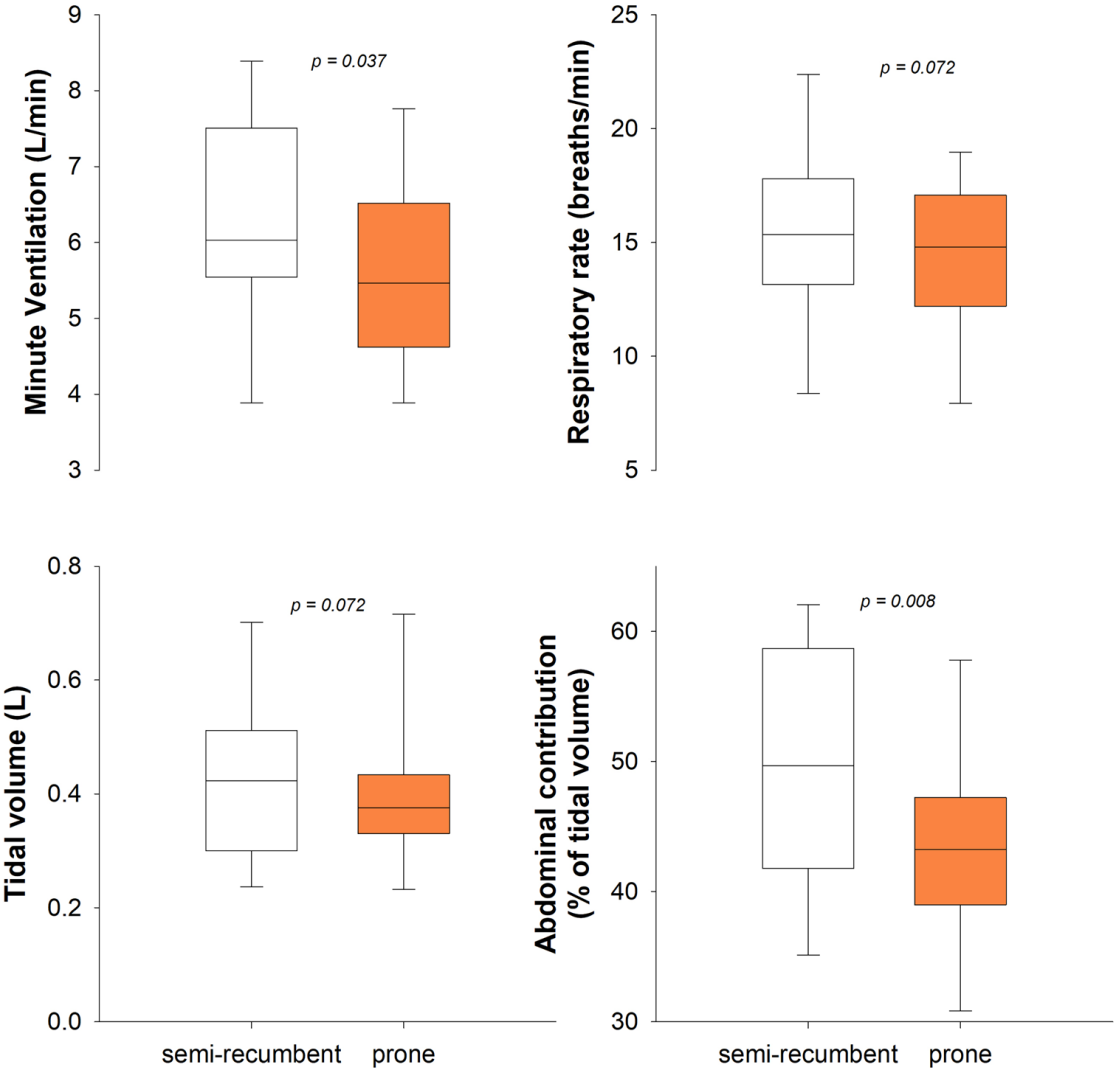
Box-plot representing the median (line within the box), the interquartile range (box height), the 5th and the 95th percentiles (lower and upper whiskers, respectively) of the minute ventilation (top left panel), respiratory rate (top right panel), tidal volume (lower left panel) and its abdominal contribution (lower right panel) at rest in semi-recumbent (white box) and prone (orange box) position

Minute ventilation was slightly reduced in the prone position. The compartmental distribution of tidal volume became less abdominal in the prone position (Fig. 2).

While the inspiratory capacity was not influenced by posture (semi-recumbent: 3.31 (2.96–3.73) L; prone: 2.97 (2.65–3.33) L; *p* = 0.074), a significant increase in vital capacity was observed in the semi-recumbent (5.01 (4.06–5.51 L) position compared to the prone one (3.66 (3.20–3.95) L; *p* < 0.001).

### Flow Effect: 50 vs. 60 L/min

In the semi-recumbent position, minute ventilation was reduced, because of reduced tidal volume and unchanged breathing frequency, with increasing flow. There was also an effect on the operational volumes. End-expiratory chest wall volume increased passing from 50 to 60 L/min mostly due to the abdominal recruitment (0.062 (0.017–0.09) L; *p* = 0.003) and not to the ribcage (0.030 (− 0.04–0.14) L; *p* = 0.147).

**Fig. 3:**
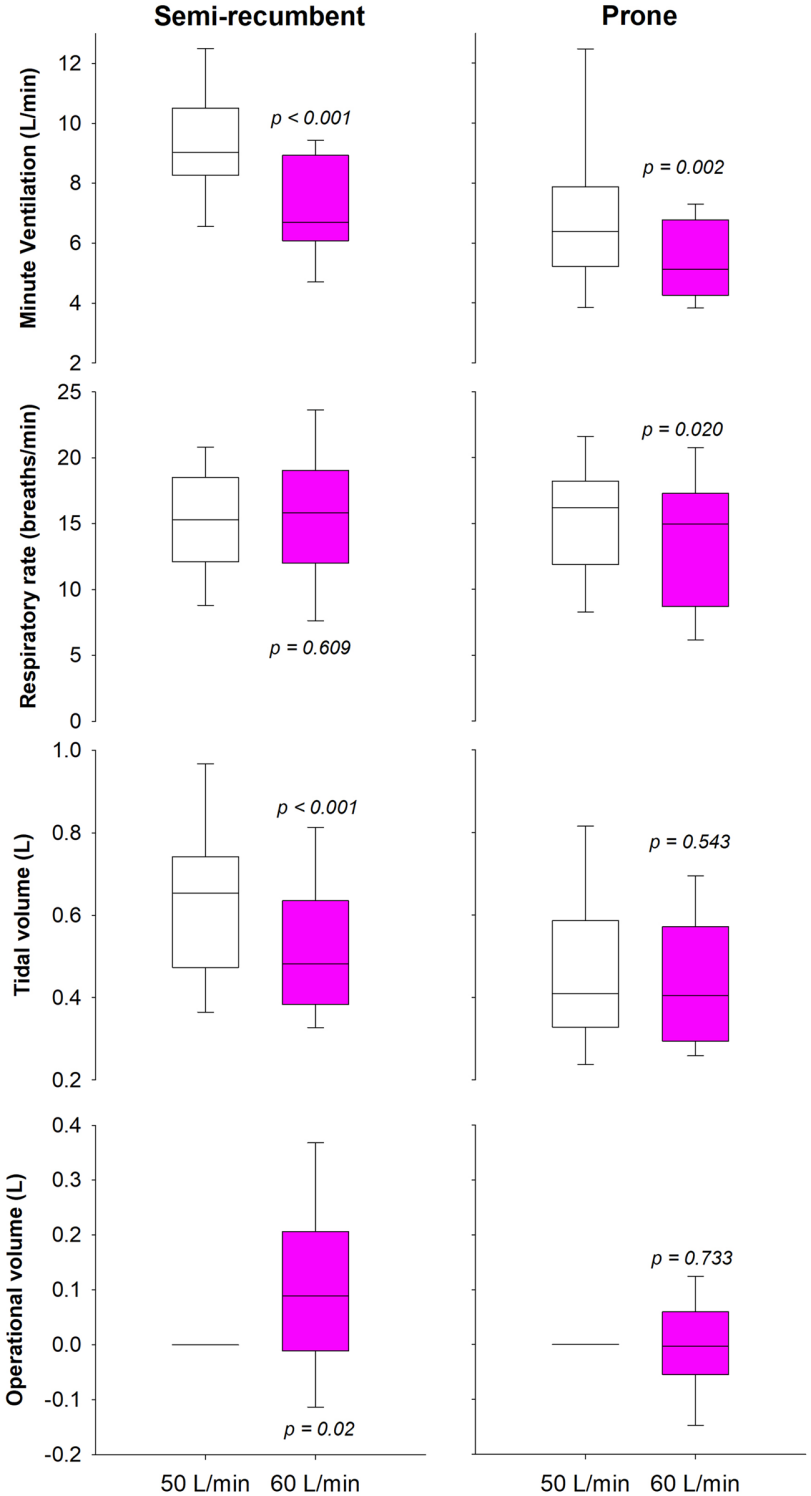
From top to bottom, box-plot representing the median (line within the box), the interquartile range (box height), the 5th and the 95th percentiles (lower and upper whiskers, respectively) of the minute ventilation, respiratory rate, tidal volume and end-expiratory chest wall volume variation in semi-recumbent (left panels) and prone (right panels) position with 50 (white box) and 60 L/min (pink box) of continuous flow

The increased flow made minute ventilation decrease also during pronation. In this position, however, ventilation changed because of the respiratory rate and no effect was found on the operational volumes (Fig. 3).

### PEEP Effect: 0 vs 5 vs. 10 cmH_2_O

**Fig. 4:**
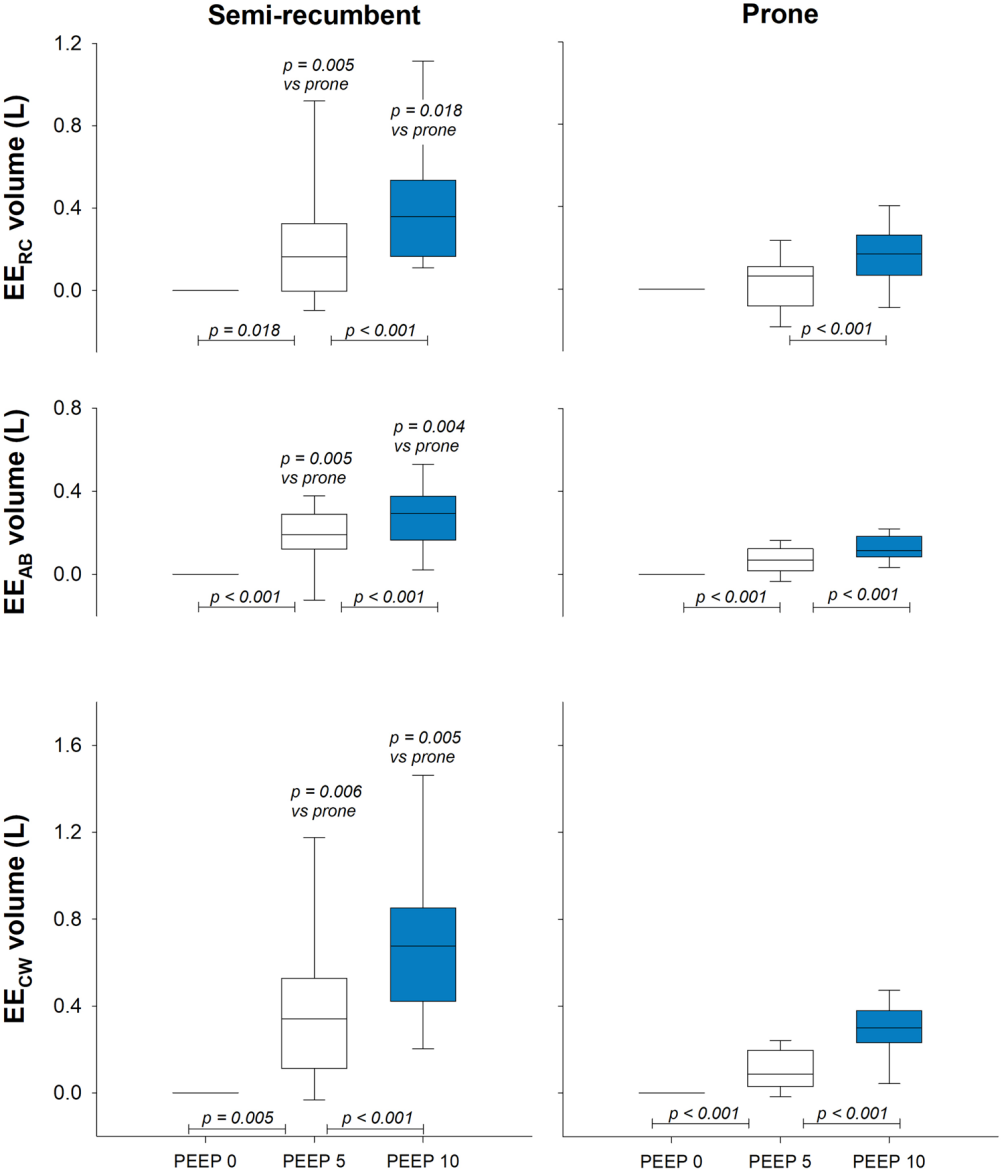
Box-plot representing the median (line within the box), the interquartile range (box height), the 5th and the 95th percentiles (lower and upper whiskers, respectively) of the end-expiratory ribcage volume variations (top panels), the end-expiratory abdominal volume variations (middle panels) and the end-expiratory chest wall volume variation (bottom panels) in semi-recumbent (left panels) and prone (right panels) position during positive end-expiratory pressure of 0 (black line at zero), 5 (white box) and 10 (blue box)

PEEP change poorly affected the ventilatory and compartmental pattern (Table 1), whereas the effects on the operational volumes are reported in Fig. 4. Except for the ribcage in prone position during the first PEEP variation (i.e., from ZEEP to PEEP = 5 cmH_2_O), a significant increase in the total and compartmental end-expiratory volumes was found in both postures and PEEP changes.

**Table 1:** Ventilatory and thoraco-abdominal pattern during the three levels of PEEP

	ZEEP			PEEP = 5 cmH_2_O			PEEP = 10 cmH_2_O			*p-*Value ZEEP vs PEEP 5	*p-*Value PEEP 5 vs PEEP 10
	Median	Q1	Q3	Median	Q1	Q3	Median	Q1	Q3		
Minute ventilation (L/min)	7.00	5.68	7.59	6.71	6.14	8.76	6.54	6.13	7.38	0.346	0.442
Breathing frequency (breaths/min)	16.03	13.99	17.96	15.81	12.43	18.57	16.10	12.07	19.07	0.865	0.393
Tidal volume (L)	0.45	0.33	0.51	0.48	0.39	0.61	0.44	0.35	0.57	0.054	0.304
Abdominal contribution to tidal volume (%)	52.00	41.71	59.44	43.04	29.50	54.78	46.68	35.24	51.86	0.003	0.609
Minute ventilation (L/min)	6.09	4.65	6.68	5.12	4.27	6.71	5.01	4.26	6.67	0.347	0.799
Breathing frequency (breaths/min)	14.83	14.25	16.71	14.94	9.44	16.93	15.27	12.11	16.78	0.495	0.369
Tidal volume (L)	0.39	0.35	0.42	0.40	0.30	0.56	0.36	0.28	0.45	0.199	0.004
Abdominal contribution to tidal volume (%)	42.48	38.65	46.48	39.17	36.19	43.85	41.01	34.23	43.88	0.059	0.468

*PEEP* positive end-expiratory pressure, *Q1* first quartile, *Q3* third quartile, *ZEEP* zero end-expiratory pressure

## Discussion

This was the first successful in vivo assessment of tidal volume during non-invasive respiratory support with H-CPAP without using a turbine-driven ventilator (and therefore in clinical mode). Although the study was performed on healthy subjects, it simulated clinical settings considering the two postures mostly used, i.e., semi-recumbent and prone, two levels of continuous flows and three of PEEP. At baseline, the prone position per se made the subject ventilate less and restricted the abdominal expansion. During CPAP, the prone position limited the recruitment of the operational chest wall volumes compared to the semi-recumbent position. Independently on posture, increased constant flow made subjects ventilate less. In addition, in the semi-recumbent position, higher flow also had a slight PEEP effect.

NIRS is frequently used in the treatment of several forms of acute or chronic respiratory failure, with the advance of absence of an endotracheal or tracheostomy tube. A crucial point for successful NIRS is the interface to deliver effective ventilation to patients without intubation [12]. There are five different types of interface: full face masks that enclose both mouth and nose, nasal masks, nasal pillows or plugs directly inserted into the nostril, mouthpieces, and helmets. The latter experienced increased attention during the COVID-19 pandemic, although helmets have been in use since the early 2000s [13–16].

A helmet is a soft, non-extensible transparent hood that fits over the subject’s head with a soft and extensible collar that fits gently around the patient’s neck. The helmet is anchored with stripes around the underarm of the subject.

The advantages of helmets are tolerability, cost-effectiveness, an excellent and gentle sealing capability that minimizes leaking and the risk of soft tissue injury. The greatest drawback is the risk of possible CO_2_ rebreathing (in case of inadequate flow), the noise and the impossibility of measuring tidal volume [17] without using a turbine-driven ventilator [18, 19]. The importance of monitoring tidal volume is preventing the risk of P-SILI. Indirect clinical signs of excessive drive (excessive breathing rate, hypocapnia, and respiratory muscle fatigue) are used instead.

The intrinsic problem of tidal volume measurements during H-CPAP is the amount of air compression/expansion (due to expiratory/inspiratory efforts) within the helmet that cannot be measured by a flowmeter. Of note, when the inspiratory flow exceeds the inflow, the patient can still receive flow from the helmet compression volume. However, this volume cannot be computed by a simple outflow trace measurement. A technique to measure tidal volume during continuous-flow helmet CPAP was recently proposed. The authors found an average relative error of − 1 ± 4.4% between the outflow signal and the reference [17]. However, these are in vitro data on a bench model. In vivo data are therefore needed. We have used OEP because it provides accurate measurements of tidal volume and because it was previously shown to be compatible with bedside non-invasive volume measurement in ICU environment [20, 21], in neonatal ICU [22, 23] and compatible with different clinical settings (postures, flow, and PEEP). OEP is neither influenced by the gas compression/expansion inside the helmet, nor affected by drifting problems (as the flowmeter does). OEP provides thoraco-abdominal volume variations, surrogates of the action of the different respiratory muscles. OEP allows the dynamic and continuous monitoring of end-expiratory chest wall volume variations. End-expiratory chest wall volume variations accurately reflect PEEP-induced changes of end-expiratory lung volume changes in mechanically ventilated paralyzed patients [24]. In addition, OEP provides highly accurate measurement of volumes in prone positions, typically adopted in critically ill patients, without requiring connection to the patient [9].

The physiological rationale of the prone position is to recruit dorsal lung units and to improve the ventilation/perfusion relationships. The latter is achieved by increasing the homogeneity of the ventilation throughout the lung. This homogeneity is affected by the gravitational effects on the lungs and by the structural variations within lung tissue [25]. At baseline, the prone position slightly reduced the minute ventilation of our healthy subjects, presumably because of a slight reduction of both breathing frequency and tidal volume. The prone position had some important constraint effects: (1) on the vital capacity; (2) on the abdominal breathing contribution (presumably because the bed and the pelvis bones limit the expansion of the abdomen); and (3) on the recruitment. As expected, the operational volumes significantly increased with increasing levels of PEEP. However, this recruitment effect was lower in the prone position compared to the semi-recumbent one. The ribcage compartment was recruited only at higher PEEP. The flow affected both the ventilatory pattern and the operational volume. Passing from 50 to 60 L/min, minute ventilation decreased. This reduction was due to lower tidal volume in the semi-recumbent position and lower respiratory rate in the prone position. Minute ventilation is linked to the washout of carbon dioxide produced by the subject inside the helmet. We can therefore speculate that as the flow increased, the washout of carbon dioxide also increased, reducing the need for ventilation.

Flow also affected the operational volume. Only in the semi-recumbent position, end-expiratory chest wall volume slightly increased (~ 100 ml) passing from 50 to 60 L/min. This result is in line with high-flow nasal cannula therapy, which is known to generate a PEEP effect. This therapy is an oxygen supply system capable of delivering up to 100% humidified and heated oxygen at a flow rate of up to 60 L/min [26]. The prone position seemed to hinder this recruitment effect of high flow, as we found no differences in end-expiratory chest wall volume.

Taken together, these results suggest that it is possible to accurately measure tidal volume during the use of H-CPAP using OEP during different clinical settings: different postures, flows, and PEEP. Each setting had specific effects on volume expansion and recruitment. Prone position limited vital capacity, abdominal expansion, and chest wall recruitment. The flow of 60 L/min reduced the need of the subject to ventilate while having a slight recruitment effect (100 mL). Higher PEEP led to progressive increasing chest wall (lung) recruitment.

The main limit of these results was that they referred to healthy lung and healthy chest wall. Efficient mechanical ventilation requires both the lung and the chest wall to be compliant. A variety of causes put patients in need of mechanical support. These include both infectious and non-infectious triggers that can injure the lung directly or indirectly due to local or systemic inflammation. The most common non-infectious causes (i.e., pancreatitis, aspiration of gastric contents, and severe traumatic injuries with shock and multiple transfusions) impact also the mechanics of the chest wall [27–29].

For these reasons, it is hard to extrapolate our results to patients whose lungs or chest wall, being damaged, might respond differently to positioning and/or incremental flows and/or PEEP. In addition, many confounding factors are frequently present in the clinical scenario. These comprise co-morbidities or the use of different devices (PEEP valves, helmet models, ventilators). Measurements on patients are therefore urged. OEP was already shown to be suitable for ventilated patients in sub intensive and intensive care units [20, 21], in operation rooms [30, 31], and with other open ventilatory systems [32, 33]. We have now shown OEP be to measure accurately the ventilatory pattern and the operational volumes also during H-CPAP. OEP can be used as the gold standard to validate emerging, non-invasive, wearable, and accurate device for volume measurement. In addition, OEP is an adequate method for patients with neuromuscular disorders, whose ventilatory pattern might be characterized by thoraco-abdominal asynchronous or even paradoxical movement. The main consequence of paradoxical breathing is inadequate air movement in and out of the lungs, despite the respiratory muscular effort. According to the disease, each compartment can move paradoxically. OEP is able to describe, quantify, and track different pathological thoraco-abdominal patterns. When the disease affects the ribcage (i.e., Osteogenesis Imperfecta [34, 35]) or the thoracic muscles (i.e., Spinal Muscle Atrophy [36]), the pulmonary ribcage moves inward during inspiration. When the disease affects the diaphragm (i.e., Amyotrophic Lateral Sclerosis [37], Duchenne Muscle Dystrophy [38], Type II glycogenosis [39]), the abdomen moves inward during inspiration. Because OEP does not need volitional manoeuvres to detect asynchronous or paradoxical breathing, it is particularly indicated to evaluate non-collaborative subjects like children [40–42].

**Fig. 5:**
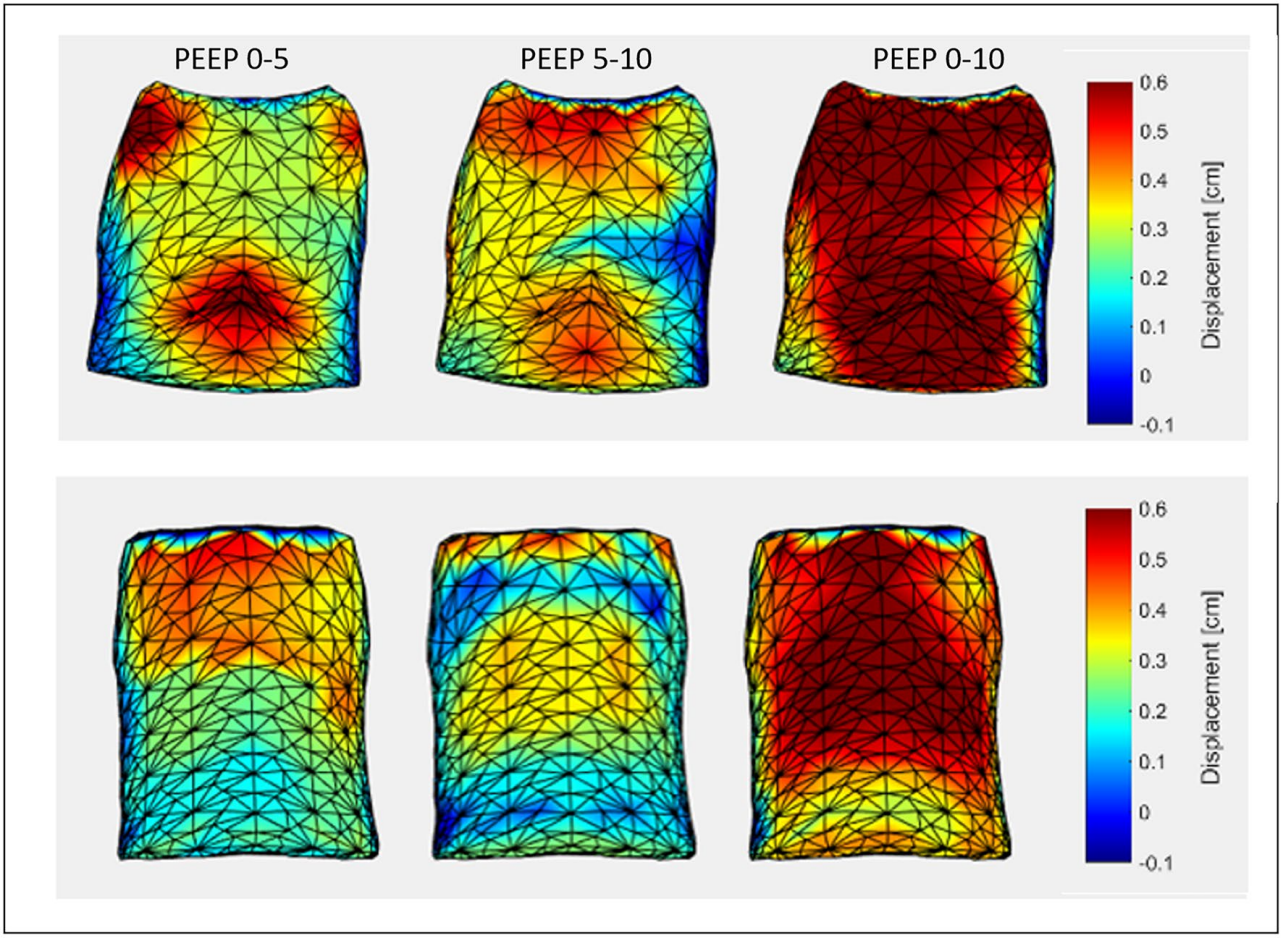
Representative heat maps of the local displacement from baseline to PEEP 5 cmH_2_O, from PEEP 5 to 10 cmH_2_O, from baseline to 10 cmH_2_O in the semi-recumbent (top) and prone (bottom) position. This is an immediate visualization of the different areas of the chest wall recruited in relation to different PEEP changes. The outward (positive) movement was displayed in red, whereas blue areas represented inward (negative) displacement (expressed in centimetres)

As shown in the exemplificative Fig. 5, it is also possible to create realistic 3D models of local trunk surface movements. This is an intuitive method to visualize and localize areas of altered expansion and/or recruitment [43]. Figure 5 referred to healthy subjects and therefore it does not evidence particular alterations. However, this graphical system was proved to localize and quantify asymmetric [43] or paradoxical [44] chest wall expansion in clinical cases. Appling this method to patients would test the relationship between the “external” movement of the chest wall and the condition of the “internal” damaged lung/chest wall.

Another limitation of the study was the time of acquisition. The results referred only to the acute phase after changes, while patients spend hours or days under the helmet.

The strengths of the study are (1) the measurement of tidal volume; (2) the simulation of clinical settings and (3) the protocol design. Indeed, we have considered levels of flow and PEEP that are commonly used for the care of mild-distress patients. The protocol was designed to investigate only the effect of the parameter that was changed. The analysis of postural effect was performed only during baseline condition, to have only the influence of posture. The analysis of flow change was performed at the same PEEP level so that no influences other than the flow would be present.

To conclude, the tidal volume, the ventilatory pattern, the thoraco-abdominal pattern and the variation of the operational volumes of the chest wall during non-invasive respiratory support with the helmet as interface were successfully measured for the first time without using a turbine-driven ventilator. The prone position seemed to reduce the need for ventilation, while limiting the recruitment effect of PEEP. Higher flow was associated with less need to ventilate and to a slight PEEP effect (but only in the semi-recumbent position).

Although these results referred to healthy subjects, they enforce the potential clinical translational value of opto-electronic plethysmography in sub-intensive and intensive units.
